# O03 The effect of antibiotic selection on collateral susceptibility and evolvability of uropathogenic *Escherichia coli*

**DOI:** 10.1093/jacamr/dlad077.003

**Published:** 2023-08-02

**Authors:** Alasdair T M Hubbard, Beth James, Rachel Whelan, Conor Meehan, Maria Rosa Domingo Sananes

**Affiliations:** Department of Biosciences, Nottingham Trent University, Nottingham NG11 8NS; Department of Biosciences, Nottingham Trent University, Nottingham NG11 8NS; Department of Biosciences, Nottingham Trent University, Nottingham NG11 8NS; Department of Biosciences, Nottingham Trent University, Nottingham NG11 8NS; Department of Biosciences, Nottingham Trent University, Nottingham NG11 8NS

## Abstract

Urinary tract infections (UTIs) are a common infection in healthy adult women and as such account for 1%–3% of all medical consultations and the second most common reason for prescribing antimicrobials in the UK. *Escherichia coli* is a frequent aetiological agent of uncomplicated UTIs, causing 75% of cases, associated with recurrence of infection (40%) and a major source of bacteraemia (∼40%). Trimethoprim and nitrofurantoin are currently recommended as first-line antibiotics for the treatment of uncomplicated UTIs. However, 48700 deaths in Europe in 2019 have been associated with antimicrobial-resistant UTIs and resistance to trimethoprim in community-acquired UTIs caused by *E. coli* is high (33.4%). With little to no antimicrobial development in recent years, new strategies are therefore required to maintain efficacy in currently available antibiotics. One such strategy is to use evolutionary principles to understand how antimicrobial resistance (AMR) to one antibiotic can affect the activity and probability of resistance development to a second antibiotic. Effect on antimicrobial activity can be assessed through collateral susceptibility, where prior resistance to one antibiotic either increases or decreases susceptibility to a second, unrelated class of antibiotic. While the probability of resistance development can be assessed by the mutant selection window (MSW), an antibiotic concentration range where AMR mutations can be selected for. This ranges from the MIC (the lowest concentration to select for resistance) to the mutant prevention concentration (MPC, the highest concentration to select for resistance). In this study, we aimed to determine the effect trimethoprim resistant derivatives of three clinical strains of uropathogenic *E. coli* on collateral susceptibility and MSWs to nitrofurantoin. We found that trimethoprim-resistant mutants conferred the expected decrease in susceptibility to trimethoprim and an increase in the MSW. This suggests prior selection with trimethoprim increases the probability of treatment failure and the selection of further mutations conferring high-level resistance to trimethoprim. We also observed collateral resistance to nitrofurantoin in the trimethoprim-resistant mutants. Importantly, however, there was a reduction in the MPC, and therefore a narrowing of the MSW, reducing the probability of selection of higher levels of nitrofurantoin resistance. This suggests that using nitrofurantoin after trimethoprim may reduce the probability of developing nitrofurantoin resistance compared with using nitrofurantoin first. These data provide a potential evidence base to use evolutionary principles when designing antimicrobial regimens to limit AMR development and improve treatment success.

